# Seroprotection of Hepatitis B Vaccine in Dental Students Two Decades after Infant Immunization and the Possible Need for Revaccination

**DOI:** 10.1055/s-0042-1743151

**Published:** 2022-04-18

**Authors:** Hanadi S. Lingawi, Ibtesam K. Afifi

**Affiliations:** 1 Preventive Dentistry Department, Faculty of Dentistry, Umm Al-Qura University, Makkah, Saudi Arabia; 2 Basic and Clinical Oral Sciences Department, Faculty of Dentistry, Umm Al-Qura University, Makkah, Saudi Arabia; 3 Department of Medical Microbiology and Immunology, Faculty of Medicine, Tanta University, Tanta, Egypt

**Keywords:** hepatitis B, antibody, immunization, dental, vaccine, Saudi Arabia

## Abstract

**Objectives** This study aimed to assess hepatitis B surface antibody (anti-HBs) persistence among dental students two decades after infant vaccination and immune response after revaccination or booster dose in nonimmune groups.

**Materials and Methods** A cross-sectional study was performed using laboratory reports for anti-HBs performed to dental students at the Umm al-Qura University from 2016 to 2020. Reports were classified according to baseline antibody titer into group I (titer <10 mIU/mL), group II (titer 10 to <100 mIU/mL), and group III (titer ≥100 mIU/mL). The basal antibody titer of each group was correlated to student's gender and birth year and compared with postrevaccination or booster dose titer in groups I and II.

**Statistical Analysis** Data of baseline and postrevaccination anti-HBs antibody titers were analyzed using Statistical Package for Social Science (SPSS). One-way ANOVA was used to compare between different means for antibody titers of students as well as baseline and postrevaccination antibody levels for nonimmune students with nonprotective antibody titers. Pairwise multiple comparison of the difference between baseline and postrevaccination anti-HBs antibody titers were done by post hoc Tukey's honestly significant difference (HSD) test. Chi-squared test was used for comparing between the frequencies. A *p* -Value of ≤0.05 was considered significant and *p* < 0.01 was considered highly significant.

**Results** A significant percentage (73.6%) of students had antibody titer <10 mIU/mL, while only 4.8% had antibody titer ≥100 mIU/mL with nonsignificant difference between both genders ( *χ* ^2^ =3.784, *p* = 0.151). A statistically nonsignificant difference was also found between the three categories of anti-HBs antibody titers among students with different birth dates ( *χ* ^2^ = 13.817, *p* = 0.182). After revaccination of nonimmune students, 100% of them showed strongly protective antibody titers with mean of 842.88 to 844.58 mIU/mL. A highly significant difference was observed between the mean baseline and postrevaccination antibody titers in both genders, with a higher mean of post revaccination ( *p* = 0.000).

**Conclusion** Two decades after infant immunization, a significant percentage of dental students failed to maintain the anti-HBs seroprotective titer. So titer measurement should be made compulsory before they begin their clinical training and revaccination or booster dose should be given to nonimmune students to maintain a high protection level.

## Introduction

Hepatitis B is a major public health problem all over the world. Health care professionals (HCP), whether they live in an endemic region or not, are considered high-risk groups to this disease. 1 Consequently, dental professionals constitute one of the most vulnerable HCP to infection by such diseases due to their direct contact with blood and saliva as well as their high rates of contact with contaminated sharp equipment. 2

In the 1970s, a vaccine against hepatitis B virus (HBV) was developed and it has been widely distributed since 1986. It is considered the main preventive method against hepatitis B and is reported to be highly reliable, accurate, and safe. Among dental practitioners, HBV vaccination is not only a self-care practice but also the most effective measure to protect patients' health, reducing the incidence of the disease in their communities. 3

A hepatitis B vaccine program was started in Saudi Arabia in 1989. Since then, Saudi Arabia implemented a national strategy for the elimination of HBV infection by applying the universal administration of HBV vaccine to all infants. Furthermore, Saudi government conditioned the issuance of birth certificates upon completion of the first-year vaccination program to confirm the maximum coverage of vaccination against HBV. The vaccination regimen established include administration of the first dose of vaccine at birth, the second at 1 month of age, and the third at 6 months of age. 4

Implementation of infant vaccination program in Saudi Arabia resulted in significant improvements in the past 30 years in terms of decrease in HBV prevalence from 6.7% to be around 1.3%. Regarding different age groups, in spite of the low HBV infection rates in the younger Saudi populations, its prevalence in older people has not decreased, which emphasizes the effectiveness of infant vaccination programs. 5 6 7

Although the effectiveness of the hepatitis B vaccination is evident, the duration of protection after vaccination is not exactly known in addition to the fact that some people remain nonresponders to vaccine and do not develop adequate anti hepatitis B surface antigen titer (anti-HBs) essential for protection against HBV. 8 9

As determination of anti-HBs response after completion of infant vaccination is not in practice in Saudi Arabia, the aim of this study was to assess the seroprotection of hepatitis B vaccine in dental students at the Umm Al-Qura University two decades after infant immunization and also to determine the immune response after adult revaccination among students with nonprotective antibody level.

## Materials and Methods

A cross-sectional study was conducted on the laboratory reports of dental students' anti-HBs antibody levels. Data were collected from the database of the Infection Control Unit, Faculty of Dentistry, Umm Al-Qura University, including student's gender, birth year, and baseline anti-HBs antibody levels as well as postrevaccination levels for students who have a nonprotective baseline level. The reports included were for students of preclinical training who received a full course of infant vaccination for hepatitis B and did not receive a booster dose the following years. Laboratory reports of students whose infant immunization records were not available or those with immunocompromised disease were excluded from the study. Reports that were performed in laboratories other than the Umm Al-Qura university medical center or showing nonquantitative antibody levels were also excluded. Measurement of HB antibody levels at the university medical center was performed by the ELIZA technique using the VITROS Anti-HBs Quantitative Reagent Pack and the VITROS Anti-HBs Calibrators on VITROS ECi/ECiQ Immunodiagnostic Systems analyzer (Ortho-Clinical Diagnostics, Inc., Raritan, NJ, United States).

### Sampling Technique and Sample Size Calculation

Sampling was done by the convenience sampling technique. The sample size was calculated using the online calculator at https://www.calculator.net/sample-size-calculator.html . The total population size was 35, using a 95% confidence interval, marginal error of 5%, and population proportion of 50%; the calculated sample size was 186.

### Data Collection and Management

Data from laboratory reports available (from 2016 to 2020) were collected not showing any nominative information, identified by serial study code and initials. Two different persons performed data entry; data were transferred to statistical database directly after verification.

### Study Procedure

Students' reports were classified according to the baseline antibody level into three groups; group I: reports of students with nonreactive antibody titer (<10 mIU/mL); group II: reports of students with reactive low antibody titer (10 to <100 mIU/mL) and relatively immune; and group III: reports of students with appropriate antibody titer (≥100 mIU/mL). The antibody level of each group was correlated to student's gender and birth year. Then, antibody levels after receiving one booster dose for group II or postrevaccination for group I students were compared with their basal antibody titer (measured 6–8 weeks either after the booster dose or the 3rd dose).

### Statistical Analysis Plan

Data of baseline and postrevaccination anti-HBs antibody titers were analyzed using Statistical Package for social Science (SPSS) software version 20. One-way analysis of variance (ANOVA) was used to compare between different means for antibody titers of students as well as baseline and postrevaccination antibody levels for nonimmune students with nonprotective antibody titers. Pairwise multiple comparison of the difference between the baseline and postrevaccination anti-HBs antibody titers by post hoc Tukey's HSD test was. Chi-squared test was used for comparing between the frequencies. A *p* -value of ≤0.05 was considered significant and *p* < 0.01 was considered highly significant.

### Ethical Part and Confidentiality

The study received ethical approval from the institutional review board of the Umm Al-Qura University (IRB approval no. HAPO-02-K- 012–2021–04–648).

## Results

### Demographic Characteristics of Participants

**Fig. 1:**
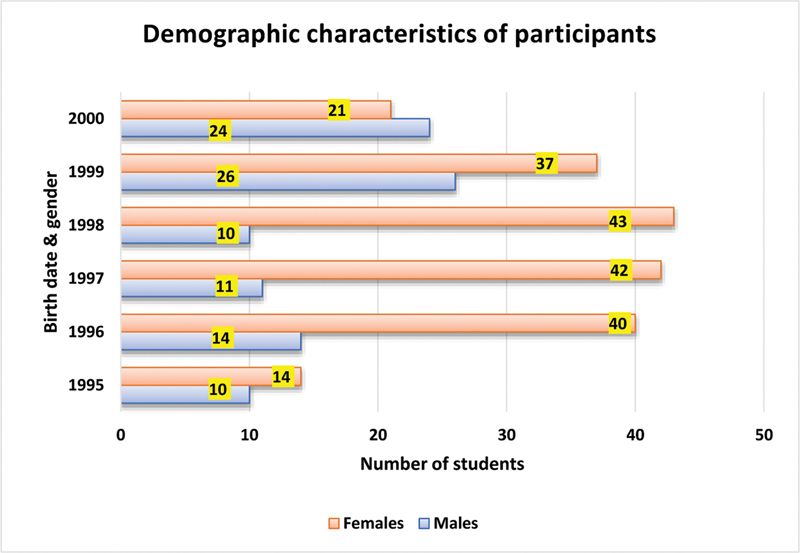
Demographic characteristics of participants.

A total of 292 laboratory reports fulfilling the selection criteria were included in the study. Female students were the majority (197; 67.5%). The least number/percentage of reports (24; 8.2%) included in the study were for students born in 1995 and the highest number/percentage (63; 21.6%) was for students born in 1999. With regard to gender, most of the male students (26) were born in1999, and most females were born in1998 (43). The number of reports for females were higher than those for males every year except the year 2000 ( Fig. 1 ).

### Categorization of Laboratory Reports according to Baseline Anti-HBs Antibody Titers in Relation to Gender

The available reports included data of 197 (67.5%) females and 95 (32.5%) males. The majority of students (73.6%) had baseline antibody titer less than 10 mIU/mL, while only 4.8% of them had antibody titer of 100 mIU/mL or more with a nonsignificant difference between both genders ( *χ* ^2^ =3.784, *p* = 0.151; Table 1 ).

**Table 1:** Distribution of baseline anti-HBs antibody titer in relation to gender

Gender	Baseline anti-HBs antibody titer (mIU/mL)			
	>10 No. (%)	10 to >100 No. (%)	≥100 No. (%)	Total No. (%)
Males	76 (80%)	17 (17.9%)	2 (2.1%)	95 (32.5%)
Females	139 (70.6%)	46 (23.4%)	12 (6.1%)	197 (67.5%)
Total	215 (73.6%)	63 (21.6%)	14 (4.8%)	292 (100%)
*χ*^2^ ( *p* -value)	3.784 (0.151)			

Abbreviations: No., number of subjects.

Note: *p* -Value is significant at ≤0.05.

### Categorization of Laboratory Reports according to Baseline Anti-HBs Antibody Titers in Relation to Birth Year

**Fig. 2:**
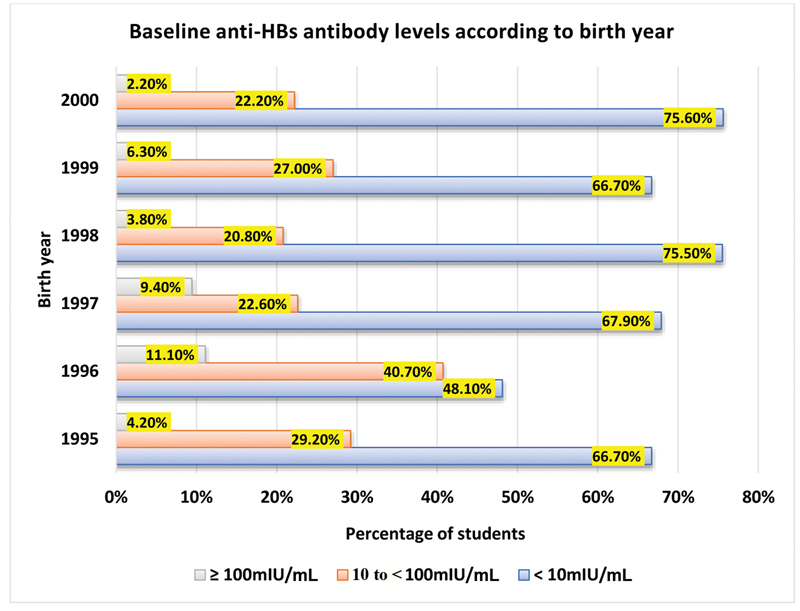
Baseline anti-HBs antibody levels according to birth year.

The highest percentages of students with nonprotective anti-HBs antibody titers (<10 mIU/mL) were those born in 1998 and 2000 (75.5 and 75.6%, respectively). For those with protective antibody titers (>100 mIU/mL), the highest percentage (11.1%) was among students born in 1996. A statistically nonsignificant difference was found between the three categories of anti-HBs antibody titers among students with different birth years ( *χ* ^2^ = 13.817, *p* -value = 0.182; Fig. 2 ).

### Anti-HBs Antibody Titers before and after Revaccination/Booster Dose of Nonimmune Students

The anti-HBs antibody titers mean was increased after revaccination in both male and female students where it increased in males from 10.08 to 844.58 mIU/mL and in females from 20.49 to 842.88 mIU/mL. Statistically, a highly significant difference was observed between mean baseline and postrevaccination antibody titers in both genders ( *p* = 0.000; Table 2 ).

**Table 2:** Means and standard deviations of baseline and postrevaccination/booster dose anti-HBs antibody titers among nonimmune students

Variable	Gender							
	Males ( *n* =95)				Females ( *n* =127)			
	Mean	SD	Min	Max	Mean	SD	Min	Max
Baseline anti-HBs antibody titer	10.08	29.55	0.00	257.00	20.49	53.14	0.00	541.46
Postvaccination/booster dose anti-HBs antibody titer	844.58	292.20	103.53	1000	842.88	280.34	123.00	1000
*F* ( *p* -Value)	800.707 (0.000 a )							

Abbreviations: Max, maximum; Min, minimum; no, subjects' numbers; SD, standard deviation.

a *p* < 0.01 highly significant.

Note: *p* -Value is significant at *p* ≤ 0.05.

### Multiple Comparison of the Difference between Baseline and Postrevaccination Anti-HBs Antibody Titers

A comparison of the differences between the mean values revealed that the mean baseline anti-HBs antibody titer was higher in female students than in male students, while the mean postrevaccination titer was higher in male students than in female students with a nonsignificant difference between both genders in either situation ( *p* = 0.977 and 1.000, respectively).

On the other hand, the mean postrevaccination anti-HBs antibody titers in both males and females was higher than the mean baseline titers with a highly significant difference between the means in both genders ( *p* = 0.000; Table 3 ).

**Table 3:** Pairwise comparison of the difference between baseline and postrevaccination anti-HBs antibody titers by Tukey's HSD test

Comparison groups	Mean difference	*p* -Value
Mean male baseline versus mean female baseline titers	−10.40850	0.977
Mean male baseline versus mean male postrevaccination titers	−834.49640	0.000 a
Mean female baseline versus mean female postrevaccination titers	−822.38991	0.000 a
Mean male postrevaccination versus mean female postrevaccination	1.69799	1.000

a *p* < 0.01 highly significant.

Note: *p* -Value is significant at *p* ≤ 0.05

## Discussion

HBV infection is still a significant public health burden in Saudi Arabia despite being the first country in the Middle East to adopt the HBV vaccine and achieving significant progress in prevention of the virus. 10 Previous studies concluded that transmission of HBV in Saudi Arabia is mainly through blood and its derivatives, hemodialysis, or by intravenous or percutaneous means. 7 Percutaneous injuries by needle sticks constitute a significant occupational hazard among dental professionals and students. 11 12 Thus, their vaccination and maintenance of adequate antibody levels is of utmost importance. Anti-HBs antibody is the serological marker measured to assess immunity induced by the HBV vaccine. 13 However, determination of the anti-HBs antibody titer is not in practice after completion of vaccination. 8

The aim of this study was to assess the persistence of anti-HBs antibody after two decades of infant vaccination among dental students and determine the immune response after revaccination of nonimmune participants.

Vaccination in Saudi Arabia was introduced in 1989 for all infants at birth, and in 1990 for all school children. 14 So laboratory reports for anti-HBs antibodies investigated in the present study were for students born from 1995 to 2000 to confirm that they received the infant HBV vaccine.

Despite the implementation of immunization programs in Saudi Arabia, timely dose at birth and three-dose coverage are estimated to be at ∼98% of Saudi infants. 15 So the infection control policy of College of Dentistry, Umm Al-Qura university, is to review the infant immunization records of students, if available, to assess their vaccination status. Many of the male students in the present study did not provide copies of these records and were excluded from the study. This explains the higher number of female participants included in the study. The lowest number of reports included in the study corresponded to students born in 1995, which may be due to the fact that our college was established in 2009, and few students had registered at that time. On the other hand, the highest percentage was for students born in 1999, which is attributed to the high total number of students registered at the college during that year.

Vaccinated students were categorized according to their anti-HB antibody titer. Those having anti-HBs antibody titer of <10 mIU/mL were considered as having nonprotective immunity, those with titer of 10 to <100 mIU/mL as having weak immunity, and those with a titer of ≥100 mIU/mL as having an appropriately high immunity. 16

In the current study, the anti-HBs baseline antibody titer was measured during the period from 2016 to 2020 after 21 years from infant immunization. Most students (73.6%) were nonprotected. This confirms the findings of previous studies that the immunity effect of hepatitis B vaccine diminishes with time. 8 17 18

In comparison with previous studies conducted on the same age group, the percentage of participants showing low anti-HBs antibody titers after 21 years from infant vaccination is lower than previously detected in Asir region, Saudi Arabia, during the period from 2012 to 2013 (88.9%) 4 and in Canada (81.5%) during the period from 2013 to 2014. 19 On the other hand, it was higher than those detected in studies performed on medical students, Taibah University, Saudi Arabia (51%), 20 medical and dental students in Malaysia (66.14%), 21 and on Chinese subjects vaccinated with plasma-derived hepatitis B vaccine (35.6%). 17

The difference in percentages in the different studies may be attributed to the number of doses received. 22 Other explanations may be deficiencies of vaccine implementation in different regions, time interval between the first and last doses of primary vaccination, and failure of vaccination in some localities that could result from improper vaccine freezing or vaccine damage. 17 Moreover, variation in vaccine brand and manufacturing company are also among the causes of differences in response, 8 where anti-HBs titer declined more rapidly among vaccinated individuals who had received recombinant rather than plasma-derived HB vaccine. 23 The decline in antibody concentration is related not only to the age at primary vaccination but also to the initial anti-HBs concentration. 24 Vaccination schedule is another contributing factor as higher anti-HBs antibody level post infant vaccination was reported when the interval between the doses was longer. 25

As there is no routine assessment of vaccine response by measuring the anti-HBs antibody titers shortly after infant vaccination, we do not know whether the students recruited with nonprotective antibody titer had previously developed antibody responses or whether they were nonresponders to the vaccine. A considerable number of these students had zero levels of anti-HBs antibodies.

In the present study, the total percentage of students showed protective antibody titers whether with low immunity titer of 10 to <100 mIU/mL or high immunity titer of ≥100 mIU/mL, support the previous assumption that the persistence of protective levels of anti-HBs antibody could happen due to adequate immune response to infant vaccination. 19

The nonsignificant difference between the baseline anti-HBs antibody titers in students with different birth years observed in the present study could be due to closely similar number of students in different years. There were nonsignificant differences in the anti-HB antibody levels between both genders, supporting the previous reports that concluded that gender has no influence on seroconversion after HBV vaccination. 22 However, higher percentages of males having nonprotective antibody titer observed could be explained by the previous finding that suggested the role of gender in the nonresponse to HBV vaccine among Asians. This was attributed to few immunological genes expressed on male Y chromosome in contrast to numerous genes on female X chromosomes that play a fundamental role in immune competence, accounting for vaccine response heterogeneity in both genders. But the authors recommend further studies to confirm this issue. 26 27

The protocol established by the infection control unit at the Umm Al-Qura university dental teaching hospital obligates students with anti-HBs antibodies titers <10 mIU/ml to be revaccinated with three doses of the HBV vaccine in accordance with the guidelines of the Centers for Disease Control and Prevention (CDC). 28 Furthermore, students with anti-HBs antibody titers of 10 to <100 mIU/ml, being a high-risk group of health care workers, are advised one booster dose of HBV vaccine according to the United Kingdom and Germany policy for health care workers. 29 These policies consider an anti-HBs titer ≥100 mIU/ml a successful vaccination.

Highly significant differences were observed between the mean baseline and postrevaccination antibody titers in both genders in the present study with higher levels reported postrevaccination. The increased antibody titer after revaccination is immunologically explained by the previous existence of humoral immune responses induced by infant vaccination. This response is generated as part of adaptive immune defenses to foreign antigens. After initial exposure to the HB antigen, B lymphocytes are activated and differentiate into plasma cells and memory B cells. Plasma cells produce antibodies specific to the antigen for clearance, and memory B cells remain for faster antibody production with future exposures to the antigen (e.g., with revaccination). 16

To accomplish long-term immunity after infant vaccination in dental students, being a high-risk group, the policy of our dental college is to revaccinate students with anti-HBs of <10 mIU/ml with three doses of vaccines and those with anti-HBs titer of 10 to <100 mIU/ml with one booster dose. By adhering to this policy, students in the present study reported 100% seroconversion of anti-HBs antibody with a highly significant difference between the mean values of pre- and postrevaccination antibody titer. This could be attributed to anamnestic response, which is defined as an increase in the anti-HBs concentration of four times or more after the booster vaccine. 28

On the other hand, different policy of giving only one booster dose for those having anti-HBs of <10 mIU/ml, followed in other countries, revealed seroconversion in lower percentages of participants. Previous studies on health care workers in Brazil and Thailand reported seroconversion only in 74.1 and 85%, respectively, with considerable percentages of participants (14.7 and 15%, respectively) still having anti-HBs antibody level <10 mIU/ml after a booster dose. 30 31

Wider scale studies on the general population in Italy and China support our policy, where 94.2 and 90% of participants, respectively, demonstrated increased anti-HBs post booster dose, while the titer remained <10 mIU/ml in 5.8 and 6.9%, respectively. 32 33 These percentages with nonresponse to a booster dose are likely explained by the possible loss of immunologic memory 17 to 18 years after primary vaccination. 34 Another important result that supports our policy is the percentage reduction of nonresponders (titer <10 mIU/ml after one booster dose) among Brazilian and Italian study participants with antibody levels decreased to 2.7 and 0%, respectively, after completing three vaccine doses. 30 32

## Conclusion

In conclusion, two decades after infant immunization, a significant percentage (73.6%) of dental students failed to maintain seroprotective antibody titer against HBV (<10 mIU/ml). But a small percentage of them still have a highly protective titer (≥100 mIU/mL). After revaccination of nonimmune students, 100% of them showed a highly protective antibody titers.

Therefore, it is strongly recommended to measure anti-HBs antibody titer in all dental students to assess their level of protection before they start clinical training. Accordingly, either one booster dose or a full course of revaccination should be administered with reevaluation of the antibody level to maintain the desired long-term seroprotection in dental students. Moreover, the threshold for hepatitis B vaccine–induced immunity in dental students should be revisited to maximize the duration of protection from occupational exposure. In addition, a national strategy to study the threshold and elucidate the immune memory after infant immunization in Saudi Arabia is recommended to assist in reducing the number of administered booster doses of vaccine.

The strengths of the present study include verification of students' HBV vaccination by vaccination records and postvaccination follow-up with quantitative anti-HBs antibody titers. The main limitations of this study are small sample size of students in one region and nonselection by random sampling, due to which the results could not be generalized to other regions. However, the consistency of our results with those from previously published studies supports the validity of these results. The other limitation is that some factors affecting immune response to vaccine such as smoking and body mass index (BMI) were not addressed.

## References

[1] Alshammari T M Aljofan M Subaie G Hussain T Knowledge, awareness, attitude, and practice of health-care professionals toward hepatitis B disease and vaccination in Saudi Arabia Hum Vaccin Immunother 2019 15 12 2816 2823 31226008 10.1080/21645515.2019.1629255 PMC6930104

[2] Garbin C AS Wakayama B Saliba T A Saliba Junior O A Garbin A JI A cross-sectional study on dental surgeons' immune status against hepatitis B virus in the Public Health System Rev Inst Med Trop São Paulo 2020 62 e18 32130358 10.1590/S1678-9946202062018 PMC7051181

[3] Nelson N P Easterbrook P J McMahon B J Epidemiology of hepatitis B virus infection and impact of vaccination on disease Clin Liver Dis 2016 20 04 607 628 27742003 10.1016/j.cld.2016.06.006 PMC5582972

[4] Ibrahim E H A booster dose for hepatitis B vaccine should be recommended by EPI in Saudi Arabia at adolescence and young adulthood Int J Virol Mol Biol 2016 5 01 8 15

[5] Saudi Association for the Study of Liver Diseases and Transplantation (SASLT) Abaalkhail F Elsiesy H AlOmair A SASLT practice guidelines for the management of hepatitis B virus Saudi J Gastroenterol 2014 20 01 5 25 24496154 10.4103/1319-3767.126311 PMC3952421

[6] Aljumah A A Babatin M Hashim A Hepatitis B care pathway in Saudi Arabia: current situation, gaps and actions Saudi J Gastroenterol 2019 25 02 73 80 30720000 10.4103/sjg.SJG_421_18 PMC6457186

[7] Abaalkhail F A Al-Hamoudi W K Khathlan A SASLT practice guidelines for the management of hepatitis B virus: an update Saudi J Gastroenterol 2021 27 03 115 126 33976009 10.4103/sjg.sjg_539_20 PMC8265399

[8] Ashraf N Hussain M U Qamar I Ashraf M Anti HBS titre; among outgoing final year MBBS students Prof Med J 2017 24 08 1167 1169

[9] Van Damme P Dionne M Leroux-Roels G Persistence of HBsAg-specific antibodies and immune memory two to three decades after hepatitis B vaccination in adults J Viral Hepat 2019 26 09 1066 1075 31087382 10.1111/jvh.13125 PMC6852111

[10] Alghamdi M Alghamdi A S Aljedai A Revealing hepatitis B virus as a silent killer: a call-to-action for Saudi Arabia Cureus 2021 13 05 e14811 34094765 10.7759/cureus.14811 PMC8170052

[11] Al-Zahrani A K Al-Sulimani R S Al-Qahtani M S Afifi I K Hepatitis B: knowledge, preventive attitude and vaccine status of dental students and interns at Umm Al-Qura University, Makkah, Saudi Arabia J Umm Al Qura Med Sci 2020 6 01 8 14

[12] Rahman B Abraham S B Alsalami A M Alkhaja F E Najem S I Attitudes and practices of infection control among senior dental students at college of dentistry, university of Sharjah in the United Arab Emirates Eur J Dent 2013 7 01 S015 S019 24966723 10.4103/1305-7456.119058 PMC4054074

[13] Sahana H V Sarala N Prasad S R Decrease in anti-HBs antibodies over time in medical students and healthcare workers after hepatitis B vaccination BioMed Res Int 2017 2017 1.327492E6 10.1155/2017/1327492 PMC5634573 29082237

[14] Yoda T Katsuyama H Analysis of antibody-negative medical students after hepatitis B vaccination in Japan Hum Vaccin Immunother 2021 17 03 852 856 32755433 10.1080/21645515.2020.1788309 PMC7993232

[15] Sanai F M Alghamdi M Dugan E A tool to measure the economic impact of Hepatitis B elimination: a case study in Saudi Arabia J Infect Public Health 2020 13 11 1715 1723 32988769 10.1016/j.jiph.2020.09.004

[16] Khoshkholgh E F Ardabili B M Habibzadeh S Evaluation of immune response towards hepatitis B virus vaccination among vaccinated students of Ardebil University of Medical Sciences in Iran Infect Epidemiol Med 2016 2 04 24 28

[17] Ma J C Wu Z W Zhou H S Long-term protection at 20-31 years after primary vaccination with plasma-derived hepatitis B vaccine in a Chinese rural community Hum Vaccin Immunother 2020 16 01 16 20 31339432 10.1080/21645515.2019.1646575 PMC7012072

[18] Gomes L C Sanson M CG Brainin P Levels of hepatitis B antibody titers are affected by age and doses gap time in children from a high endemic area of the western Amazon PLoS One 2021 16 07 e0253752 34197516 10.1371/journal.pone.0253752 PMC8248698

[19] Huynh C Minuk G Y Uhanova J Baikie M Wong T Osiowy C Serological and molecular epidemiological outcomes after two decades of universal infant hepatitis B virus (HBV) vaccination in Nunavut, Canada Vaccine 2017 35(35, Pt B):4515 4522 28736196 10.1016/j.vaccine.2017.07.040

[20] Mahallawi W Persistence of hepatitis B surface antibody and immune memory to hepatitis B vaccine among medical college students in Madinah Ann Saudi Med 2018 38 06 413 419 30531175 10.5144/0256-4947.2018.413 PMC6302994

[21] Ng K P Ngeow Y F Hepatitis B seroprevalence among university of Malaya students in the post-universal infant vaccination era Med J Malaysia 2013 68 02 144 147 23629561

[22] Sacchetto M S Barros S S Araripe TdeA Silva A M Faustino S KM da Silva J MN Hepatitis B: knowledge, vaccine situation and seroconversion of dentistry students of a public university Hepat Mon 2013 13 10 e13670 24348639 10.5812/hepatmon.13670 PMC3842515

[23] Bruxvoort K Slezak J Huang R Association of number of doses with hepatitis b vaccine series completion in US adults JAMA Netw Open 2020 3 11 e2027577 33252692 10.1001/jamanetworkopen.2020.27577 PMC7705595

[24] Anupriya A Priyanka N Ganesh V Uma A Antibody to hepatitis B surface antigen in vaccinated health care workers IJRDPLS 2016 5 05 2307 2310

[25] Afzal M F Sultan M A Saleemi A I Immune response and anamnestic immune response in children, 9 months–10 years after a 3-dose primary hepatitis B vaccination J Ayub Med Coll Abbottabad 2016 28 04 715 717 28586593

[26] Yang S Tian G Cui Y Factors influencing immunologic response to hepatitis B vaccine in adults Sci Rep 2016 6 06 27251 27324884 10.1038/srep27251 PMC4914839

[27] Trevisan A Frasson C De Nuzzo D Nicolli A Scapellato M L Significance of anti-HB levels below 10 IU/L after vaccination against hepatitis B in infancy or adolescence: an update in relation to sex Hum Vaccin Immunother 2020 16 02 460 464 31487228 10.1080/21645515.2019.1656483 PMC7062447

[28] Schillie S Vellozzi C Reingold A Prevention of hepatitis B virus infection in the United States: recommendations of the Advisory Committee on Immunization Practices MMWR Recomm Rep 2018 67 01 1 31 10.15585/mmwr.rr6701a1 PMC5837403 29939980

[29] Komatsu H Klenerman P Thimme R Discordance of hepatitis B vaccination policies for healthcare workers between the United States, the United Kingdom and Germany Hepatol Res 2020 50 03 272 282 31845478 10.1111/hepr.13470

[30] Lopes M H Sartori A MC Souza T VG Mascheretti M Chaves TdoS Hepatitis B revaccination for healthcare workers who are anti-HBs-negative after receiving a primary vaccination series Rev Soc Bras Med Trop 2012 45 05 639 642 23152350 10.1590/s0037-86822012000500018

[31] Posuwan N Vorayingyong A Jaroonvanichkul V Implementation of hepatitis B vaccine in high-risk young adults with waning immunity PLoS One 2018 13 08 e0202637 30125298 10.1371/journal.pone.0202637 PMC6101408

[32] Study Group Romanò L Galli C Tagliacarne C Persistence of immunity 18-19 years after vaccination against hepatitis B in 2 cohorts of vaccinees primed as infants or as adolescents in Italy Hum Vaccin Immunother 2017 13 05 981 985 28272974 10.1080/21645515.2017.1264795 PMC5443392

[33] Zhao Y L Han B H Zhang X J Immune persistence 17 to 20 years after primary vaccination with recombination hepatitis B vaccine (CHO) and the effect of booster dose vaccination BMC Infect Dis 2019 19 01 482 31146699 10.1186/s12879-019-4134-9 PMC6543564

[34] Katoonizadeh A Sharafkhah M Ostovaneh M R Immune responses to hepatitis B immunization 10-18 years after primary vaccination: a population-based cohort study J Viral Hepat 2016 23 10 805 811 27126365 10.1111/jvh.12543

